# The “Northwest” and the “East”

**Published:** 1904-04-15

**Authors:** 


### THE "NORTHWEST" AND THE "EAST."

(Being some observations on the dispute between the G. V. Black adherents and his critics in the East upon cavity-formation and the introduction of gold fillings; the "Northwest" championed by E. K. Wedelstaedt, of St. Paul, Minn., and the "East" by M. L. Rhein, of New York.)

The *International Dental Journal* has recently been the forum where some interesting reading upon the above subject has been given to the dental profession. Firstly, Dr. Rhein in the September issue had an article entitled "The Technique of Approximal Restorations with Gold in Posterior Teeth." In the January number, Dr. Wedelstaedt, in a "Report of the Demonstrator of the G. V. Black Dental Club of St. Paul," raked Dr. Rhein over the coals in truly Wedelstaedtian way. Now Dr. Rhein, in the March *International,* has a "Reply to Dr. Wedelstaedt," and the tournament is on. These worthy knights are perfectly capable of taking care of themselves, and as they have the "courage of their convictions," each word they say commands respectful attention. We do not champion either side of the controversy exclusively, but only desire our expressions and comments upon the statements of both to be considered as an individual opinion. When Dr. Rhein says that, "It had become a popular delusion in a certain section of the country that this (extension for prevention), was an entirely new doctrine evolved by Dr. G. V. Black," he undoubtedly states a fact. *It is a delusion;* because such shaping of cavities has practically been in use by the foremost men in many sections of our country long before the phrase "extension for prevention" was coined; *but it is equally true that to Dr. Black and his friends the credit for systematizing such practice and placing it upon a better working basis is unquestionably due.* We are not aware that Dr. Black or his friends have claimed more than this; indeed, they might as well claim that the conservation of the natural teeth by fillings inserted upon scientific lines was unknown until extension for prevention came to the fore, and every one knows they have not done such a foolish thing.

Dr. Wedelstaedt spends a lot of time saying that he never wants to hear the expression "extension for prevention" again; that the "G. V. Black Club of St. Paul" for "nearly two years has not said anything about extension for prevention." We desire seriously to ask if Dr. Wedelstaedt is not quibbling? The expression is totally unobjectionable to us, and is expressive of many essentials in making a filling perfect, and why the reiteration of the phrase is "disgusting" to Dr. Wedelstaedt passes our comprehension entirely. On the contrary, we affirm that the expression "extension for prevention" has been an immense educational force in teaching the proper formation of cavities, and that Dr. Wedelstaedt some time must confess to the same opinion.

Dr. Rhein advocates "an all-cohesive gold filling," to which Dr. Wedelstaedt strenuously objects and advises non-conhesive gold at the "gingival third." We think both are wrong. A few layers of non-cohesive gold, to make the cervical adaptation easier, is all that is necessary; but if the third on the filling is non-cohesive, the filling will sometimes give way under unusual stress. Dr. Rhein is surely correct when he says that cohesive gold can be placed at the cervical margin perfectly, but it is certainly easier and more rapidly accomplished if a few layers of the non-cohesive is used.

---

Dr. Wedelstaedt says: "Tight margins cannot, under any circumstances, be made against a smooth surface, if gold is the filling material used." Dr. Rhein says: "I consider it very objectionable to condense gold against the surface of enamel rods, if prepared by chisels, leaving a roughened surface such as he describes." Dr. Wedelstaedt endeavors to explain why gold cannot be packed against a smooth margin, but he signally fails to do so. He always will fail in the impossible.

---

Dr. Rhein says: "It might as well be stated here that it is not possible to produce ideal results in cohesive gold by the use of pellets, cylinders, ropes or any form except the regular foil or rolled gold." Dr. Rhein accuses Dr. Wedelstaedt of being "dogmatic," and declines to take his "say-so;" but Wedelstaedt never made a more dogmatic statement than the above from Dr. Rhein. Thousands of the finest operators who daily make perfectly "homogeneous solid plugs" with other forms of gold than foil or rolled gold will wonder when dental writers will cease making unwarranted statements.

---

Dr. Rhein advocates doing a larger filling in sections on account of the tedium; for instance, placing the cervical half or third, then burnishing, temporary filling the balance of the cavity with gutta-percha and completing at another sitting. Dr. Wedelstaedt says there will be "a bad joint where the filling is continued." Not necessarily so, Dr. Wedelstaedt; but, in the name of common sense, would it not be easier for the patient to conclude the operation at once? For after the cavity is shaped and the cervical third or half completed, it usually needs only a few minutes to complete the operation? And if the operation is completed subsequently, the rubber must again be placed, great care in drying and freshening the surface must be taken, and this alone will usually take as much time as it would to go on and complete the operation at the first sitting. Two bites on a cherry is very good because the cherry itself is pleasant, and we desire to prolong the satisfaction; but filling a tooth is not so excruciatingly exhilarating that either operator or patient will desire to extend the experience indefinitely. We vote NO to the installment plan. There are very many other points in the controversy between these valiant and sturdy antagonists that we think deserve criticism, but we have to conclude, and add that the admirable things both men say far out-balance that which deserves criticism.—Editorial in *Western Dental Journal.*

